# Brain amino acid sensing for organismal amino acid homeostasis

**DOI:** 10.1098/rsob.250092

**Published:** 2025-08-06

**Authors:** Anthony H. Tsang, Liubou Samson, Clemence Blouet

**Affiliations:** ^1^ Institute of Metabolic Science-Metabolic Research Laboratories and Medical Research Council Metabolic Diseases Unit at the University of Cambridge, Cambridge, UK

**Keywords:** dietary protein, amino acid, homeostasis, hypothalamus, metabolism, food intake, physiology

## Abstract

Amino acids are essential for normal physiological functions, and disruptions in their circulating concentrations are implicated in the pathophysiology of various diseases. Therefore, understanding the mechanisms that regulate circulating amino acid levels in normal physiology is of critical importance. Evidence indicates that in healthy mammals, post-absorptive circulating levels of essential amino acids are maintained within a range that varies little from day to day or following bidirectional changes in dietary protein intake. This suggests the presence of homeostatic control mechanisms. Here, we propose a conceptual framework for the homeostatic regulation of essential amino acid availability, emphasizing the role of the brain in generating feedback controls to restore baseline levels acutely after a meal and during chronic changes in dietary protein intake. We review current evidence supporting brain amino acid sensing as a component of this regulatory system, integrating peripheral and central signals to modulate dietary protein intake and peripheral amino acid metabolism. We highlight major knowledge gaps regarding the specific neural circuits, molecular mechanisms and physiological outcomes of brain amino acid sensing. Future inquiry using the proposed framework and addressing these gaps will significantly enhance our understanding of the pathways involved in the maintenance of circulating amino acid availability and the regulation of lean mass in health, disease states or in response to therapeutic strategies for metabolic diseases.

**Figure:**
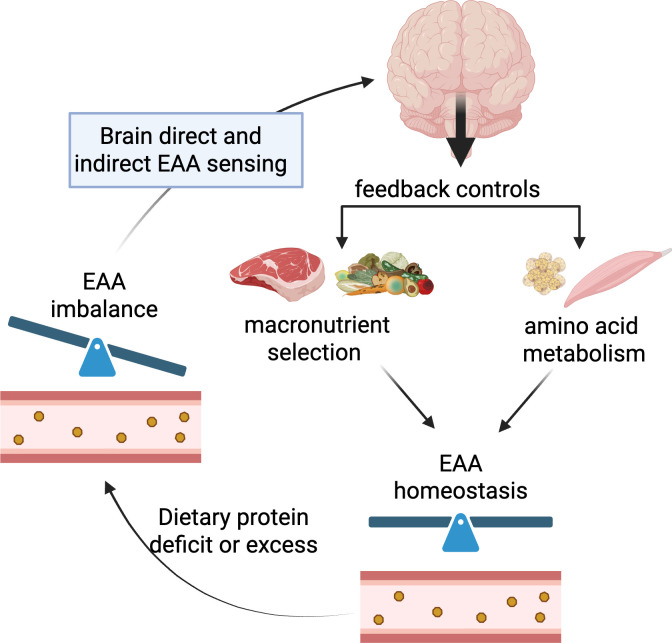

## 1. Introduction: a conceptual framework to interrogate organismal amino acid homeostasis

**Figure 1.**
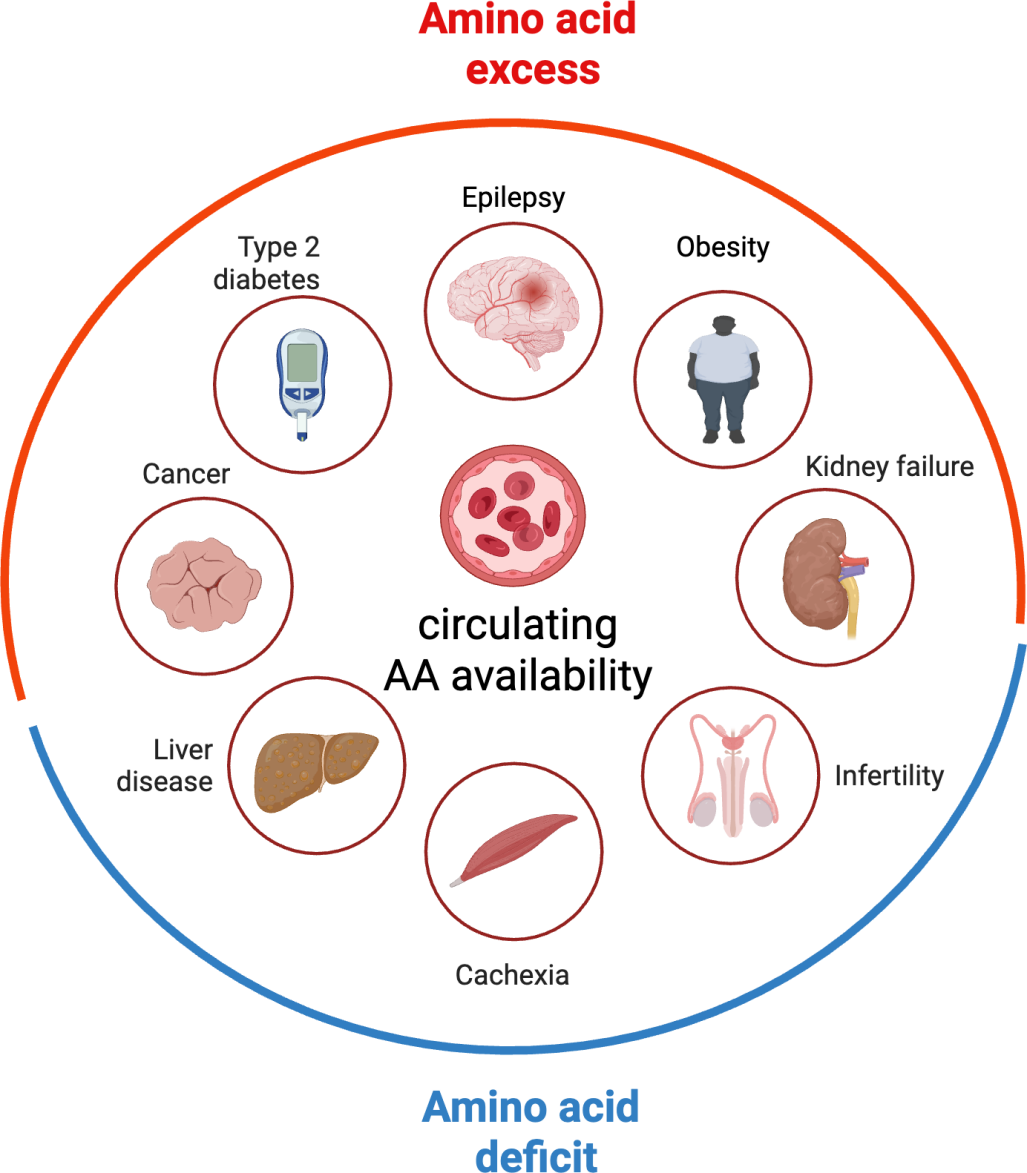
Systemic amino acid availability in health and disease. Impaired amino acid availability associates with various diseases including cancers [7], type 2 diabetes [8], epilepsy [9], obesity [10], kidney failure [11], liver disease [12], cachexia [13] and infertility [14].

Amino acids are fundamental building blocks of proteins, act as signalling molecules influencing various physiological processes and serve as precursors to key neurotransmitters and hormones. Nine amino acids—histidine, isoleucine, leucine, lysine, methionine, phenylalanine, threonine, tryptophan and valine—cannot be synthesized by mammalian cells (essential amino acids (EAAs)). After protein degradation, EAAs are recycled for new protein synthesis but are also continuously lost by oxidative catabolism. A continuous supply of dietary EAAs is required to replace these losses, in particular when anabolic needs are high such as during growth or reproduction, but even under baseline conditions and after growth has ceased [1]. On the other hand, elevated levels of EAAs cause neurological diseases, for example in maple syrup urine disease, where decreased catabolism of branched-chain amino acids (BCAAs) leads to elevated circulating and brain levels of BCAAs and neurological disorders [2]. Elevated circulating levels of BCAAs are increasingly recognized as a contributing factor to metabolic diseases such as obesity, insulin resistance and type II diabetes [3,4] and several types of cancer [5,6]. Thus, optimal physiology and health rely on the maintenance of EAA availability within a defined range to avoid both deficit and excess (figure 1).

Amino acids are not stored in a readily available manner in the body but are degraded when consumed in excess [15], unlike glucose and fatty acids, which are stored through dedicated mechanisms in the form of hepatic glycogen and lipid droplets in adipocytes. Proteins available in skeletal muscle are not stored for this purpose *per se*, as they serve important physiological functions, including muscle contraction [16]. Thus, the need to avoid EAA excess or deficit is challenged by the lack of a dedicated storage compartment for amino acids. This raises the fundamental questions of how tightly whole-body amino acid availability is regulated and through which mechanisms.

Available data support the notion that circulating levels of EAAs are regulated in healthy humans with negligible day-to-day variations [17] but also in response to acute and long-term manipulations in dietary protein intake in rodents and healthy humans [18] (table 1). For example, studies demonstrate that EAA levels are buffered after meals, returning to baseline relatively quickly, within a timeframe similar to that required for blood glucose levels to normalize postprandially [18]. This buffering also occurs in the post-absorptive state and under conditions of chronic changes in dietary protein intake (table 1). In response to dietary protein dilution, amino acid concentrations are reduced in the hepatic portal vein [19] but are maintained—or even slightly elevated—in systemic circulation due to adaptive changes in hepatic amino acid metabolism [20]. This suggests a robust regulatory mechanism ensuring amino acid homeostasis under varying dietary conditions.

**Table 1.** Effect of changes in dietary protein consumption on post-absorptive plasma amino acid concentrations and in physiological states.

reference	model		effect on circulating [AA]	
			[EAA]	[NEAA]
PMID: 25133427	mouse	low-protein feeding (5%) for 2 weeks	no change	higher
PMID: 37118349		low-protein feeding (5%): 3 h fast	no change	higher
PMID: 22568979	rat	low-protein feeding (10%) for 14 days: 8 h fast	no change	no change
PMID: 21147771		low-protein feeding (6%) for 7−10 days: 12 h fast	no change	↑ [Ser] and [Gly]
PMID: 10516129		low-protein diet (6%) for 5 weeks	all lower	most higher
PMID: 24898843		low-protein feeding (10%) for 4 days: ad libitum	↓ [BCAA] initially but normalized on day 4	↓ initially but normalized or overshot on day 4
PMID: 35267952	mouse	high-protein diet (61%) for 7 days	↑ [BCAA], [Tyr] and [Phe]	↓ [Gly]; ↑ [Med] and [Pro]
PMID: 37118349		high-protein feeding (25% or 35%): 3 h fast	no change	no change
PMID: 33910835		high-protein feeding (25%): 6 h fast	↑ [Val]	↓ [Ser]
PMID: 22568979	rat	high-protein feeding (25%) for 14 days: 8 h fast	↑ [BCAA]	no change
PMID: 10516129		high-protein diet (35%) for 5 weeks	↓ [Threo]	↑ [Met]; ↓ [Asp] [Citr] [Hist] [Ser]
PMID: 15155276		high-protein (50%) feeding for 15 days, 3 or 6 mo	no change	no change
PMID: 571951	healthy human volunteers	diurnal variations	no change	no change
PMID: 571951		low protein (0.7 g kg^−1^ d^−1^) for 7−10 days: 12 h fast	no change	no change
PMID: 571951		high protein (1.5 g kg^−1^ d^−1^) for 7−10 days: 12 h fast	no change	no change
PMID: 26395436		meat-eaters versus fish-eaters versus vegetarian versus vegans	changes in [Trp]	changes in [Lys] and [Met]
PMID: 12949358		high-protein feeding (2 g kg^−1^ d^−1^) for 16 days: overnight fast	↑ [BCAA]	↓ [Glu] [Gln] [Ala] [Gly]

How is this achieved? If circulating EAA concentrations are maintained within a narrow range between meals in healthy conditions, this suggests the contribution of homeostatic pathways, i.e. physiological mechanisms that maintain variables of the internal milieu to constant levels [21]. While mechanisms mediating cellular amino acid homeostasis are well documented [22,23], our understanding of the pathways that maintain whole-body EAA availability to support organismal amino acid homeostasis remains limited. As with all homeostatic systems, the regulation of EAA homeostasis is expected to involve sensors, error detectors that assess deviations from target values (setpoints), and control pathways that recruit effectors to restore setpoint levels [21].

In this framework, protein intake is one of the controlled effectors contributing to the regulation of EAA homeostasis, rather than the ‘defended’ variable. Of note, protein intake is not a variable of the internal milieu, which defines homeostatically regulated variables [24]. This framework also highlights the need to consider additional effectors that might be engaged to maintain whole-body amino acid homeostasis and buffer against variations secondary to changes in dietary protein intake. Several additional effector mechanisms involved in maintaining EAA homeostasis have been characterized, including gut regulation of EAA absorption, splanchnic first-pass metabolism [25,26], muscle protein synthesis [27], iBAT oxidation [28] and kidney reabsorption [29]. However, whether these effectors are coordinated to optimize amino acid homeostasis is unknown. Furthermore, our understanding of the EAA sensors crucial for EAA homeostasis and the mechanisms that establish EAA setpoints is limited.

**Figure 2.**
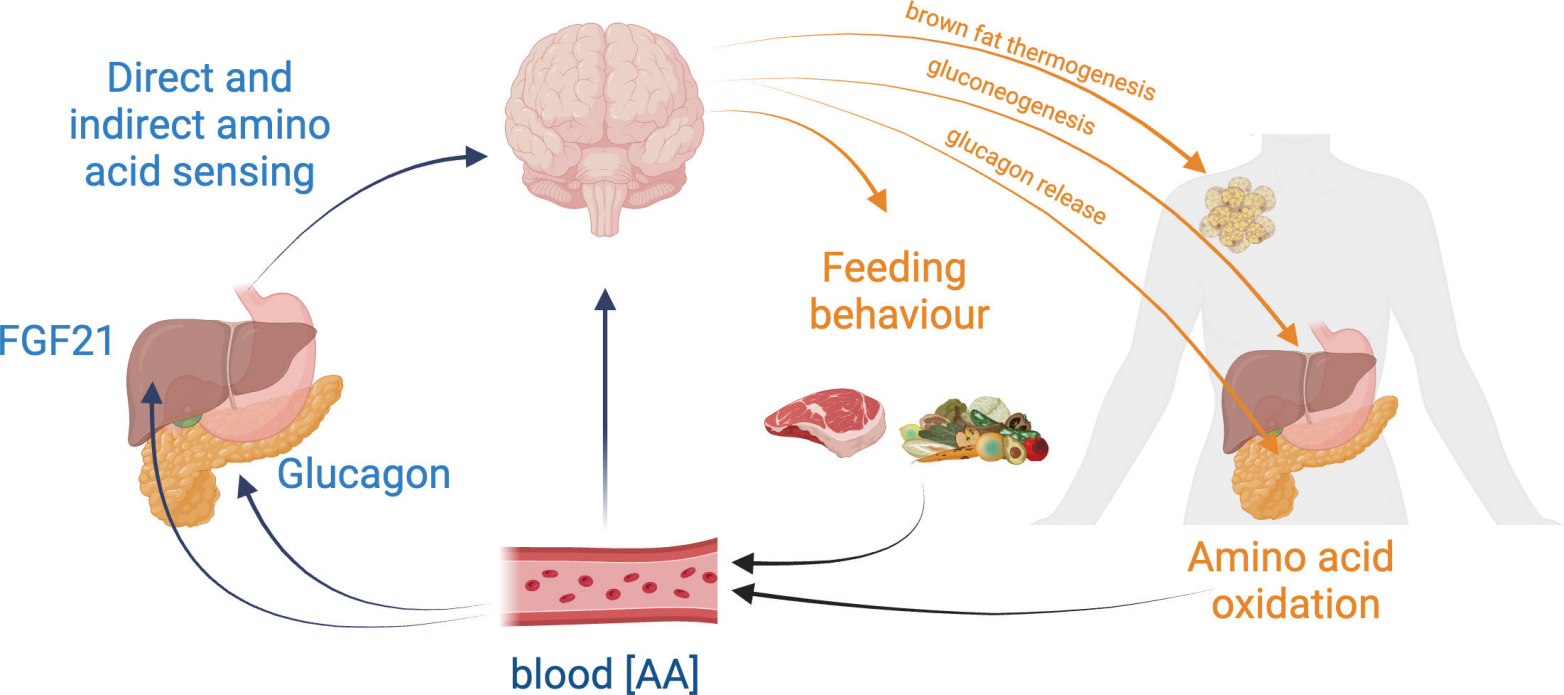
Proposed framework for the homeostatic regulation of systemic amino acid availability. Circulating amino acid concentrations are sensed by the brain either through peripheral hormones (i.e. hepatic FGF1 or pancreatic glucagon) or directly in amino acid-sensing neurons. This sensing triggers feedback responses controlling behavioural and metabolic effectors that modulate protein dietary intake and organismal amino acid utilization, promoting the maintenance of whole-body amino acid availability.

The brain plays a key role in homeostatic processes including the regulation of energy and nutritional homeostasis [30]. Beyond its requirement for the expression of behavioural responses that control dietary nutrient intake, specialized brain regions such as the hypothalamus recruit multiple peripheral effectors to maintain homeostasis via neuroendocrine and autonomic pathways. In this review, we will discuss the available literature on brain amino acid sensing in the control of feeding and metabolic responses to altered levels of dietary protein intake or systemic amino acid availability (figure 2). We will discuss how existing data inform on the putative role of the brain in whole-body amino acid homeostasis, highlighting controversies and knowledge gaps. We will first focus on the effects of brain direct and indirect amino acid sensing on feeding responses promoting organismal animal acid homeostasis. We will then examine the potential role of these pathways in the control of metabolic effectors contributing to amino acid homeostasis. Last, we will explore the role of brain amino acid sensing in extreme dietary situations associated with amino acid deficit, where functional trade-offs emerge to promote short-term survival.

## 2. Brain amino acid sensing and the control of feeding behaviour

Variations in dietary nutrient intake and systemic nutrient availability are detected by gastrointestinal and hepatic sensors, which relay this information to the brain via neurohumoral routes. In addition, specialized brain nutrient-sensing neurons directly detect changes in circulating nutrient levels. In this first section, we will review our current understanding of the adaptive feeding responses produced by changes in dietary protein intake and the contribution of indirect and direct brain amino acid sensing mechanisms in these responses.

### 2.1. Adaptative feeding behaviours to changes in dietary protein intake

Variations in dietary protein intake elicit adaptive changes in feeding behaviour, which mitigate the impact of these dietary changes on internal amino acid availability (see [31] for review). We will first review how feeding behaviour adapts to changes in dietary protein intake, the amino acid sensing mechanisms that have been proposed to mediate these responses, and the brain pathways recruited to mediate the expression of these behaviours.

Using the macronutrient self-selection paradigm, where animals can freely choose between pure macronutrients or multiple diets with complementary macronutrient ratios, multiple studies reported that rodents freely select a diet with a fixed protein content, which they maintain over days [32–35]. This indicates that mammals can adjust their dietary choices to the available food sources and, when possible, maintain dietary protein intake at a fixed level, often referred to as ‘protein intake target’ [36]. This fine-tuning occurs within hours, suggesting the rapid contribution of post-absorptive sensors. Likewise, healthy human participants can maintain their protein intake remarkably constant within the context of a meal, under conditions where energy, carbohydrate and fat intake vary significantly [37–39]. Thus, the direct control of protein intake might be a primary strategy to avoid EAA excess or deficiency and achieve EAA homeostasis.

The second line of evidence supporting the contribution of feeding behaviour in the maintenance of EAA homeostasis is the effect of dietary protein content (in the absence of macronutrient self-selection, i.e. when only one diet is available) on ad libitum energy intake. Strong evidence shows that rodents increase their energy intake in response to protein dilution and decrease it when consuming protein-rich diets [40–46]. This phenomenon, known as protein leverage [47], was first observed in invertebrates and later extended to non-human primates, therefore showing evolutionary conservation [48]. The classic interpretation is that the protein-dependent control of energy intake helps control protein consumption, aiming to meet the ‘protein intake target’ [36]. Similarly, in humans, higher protein intake reduces ad libitum energy intake [39,45,49–53], and a few studies report a hyperphagic response to protein restriction in humans [54]. In both rodents and humans, compensatory hyperphagia during protein restriction does not extend ad infinitum and ceases below dietary protein concentrations associated with severe protein malnutrition [52,55–57]. In addition, this mechanism does not fully compensate for changes in dietary protein concentration. Both rodents and humans rarely maintain dietary protein intake during experimental dietary protein excess or deficit [52]. This raises the question of the contribution of additional mechanisms to maintain EAA homeostasis under these conditions. It also points to a trade-off between the maintenance of dietary protein intake and other competing systems regulating appetite and balancing energy, carbohydrate and lipid intake.

Dietary protein restriction consistently promotes protein preference in rodents [46,58–62] and increases their motivation to work for a protein reward [59,63,64]. This behaviour appears to be a strategy to optimize protein intake when dietary protein availability is persistently low. The timeframe for developing this behaviour is not well documented, as protein preference is typically measured at least one week after exposure to low-protein diets. This leaves open the question of whether protein preference serves as a primary mechanism to prevent EAA deficits or arises as a result of compromised EAA homeostasis. In contrast, many species can quickly detect and avoid diets that are severely imbalanced in amino acids or deficient in EAAs [65], an unlearned aversive response [66–68]. Rodents rapidly recognize and consume solutions providing the missing EAAs [69]. This indicates the existence of amino acid-specific sensory mechanisms that drive consummatory behaviours under these conditions. Emerging evidence indicates that a history of protein restriction produces long-term changes in protein preference, suggesting the contribution of cognitive strategies for EAA homeostasis [70,71].

In summary, it is clear that when EAA homeostasis is compromised in conditions of acute and chronic deficit or excess in dietary protein availability, the organism responds by producing a variety of behavioural responses. We propose that these responses are mediated by distinct mechanisms in the brain, recruiting different sensing mechanisms and neural circuits. In the next section, we will review our current understanding of how the brain monitors peripheral amino acid availability to elicit these responses, the feeding pathways activated in response to changes in dietary protein intake, and their overall contribution to EAA homeostasis, energy intake and energy balance.

### 2.2. Brain sensing of systemic amino acid availability: pre-absorptive sensing and vagal afferents

The central regulation of feeding behaviour integrates pre-ingestive, pre-absorptive and early post-absorptive (pre-hepatic) signals [72,73] which can both promote or inhibit consummatory behaviours. In this section, we will briefly review our current understanding of how these pathways are recruited in response to variations in dietary amino acid intake and might contribute to the maintenance of amino acid homeostasis.

Taste receptors in the taste buds detect a wide range of amino acids, triggering rapid changes in oral motor behaviour [74]. EAA deprivation leads to a learned preference for solutions containing the missing EAAs, a behaviour that emerges only after post-absorptive detection of the solution’s nutritional value [75–77]. While the underlying mechanisms remain poorly characterized, this suggests that the organismal amino acid status modulates taste-induced consummatory responses, contributing to the restoration of amino acid homeostasis, at least under conditions of severe deficit. However, no evidence currently supports the ability of mammalian taste receptors to discriminate between individual amino acids or to directly signal dietary protein levels or amino acid concentrations to the brain [78–81].

Intestinal detection of amino acids is a well-established mechanism through which high dietary protein intake promotes satiation. Amino acids are sensed by enteroendocrine cells via various G-protein-coupled receptors, including taste receptors or the calcium-sensing receptor, leading to the release of satiation signals such as cholecystokinin (CCK), GLP-1 and PYY [82–87]. While this mechanism contributes significantly to reducing energy intake in response to high-protein diets and mitigates against organismal amino acid excess, it represents a shared sensing pathway utilized by all macronutrients. Currently, there is limited evidence to suggest that enteroendocrine cells can specifically signal intestinal amino acid availability to the rest of the organism.

Recent work has revealed the molecular diversity of vagal sensory neurons and their ability to relay nutrient-specific information to the brain, both for satiation and motivated feeding [88]. Duodenum infusion of amino acid rapidly activates vagal afferents, leading, for example, to the inhibition of hunger-promoting agouti-related peptide (AGRP) neurons in the hypothalamus [89]. Recent evidence suggests that pre-absorptive amino acid sensing rapidly changes the activity of mesolimbic dopaminergic circuits in protein-deprived mice, but the exact sensing modality recruited under these conditions is unknown [64]. More work is needed to identify specific amino acid sensing mechanisms in vagal sensory neurons and their role in promoting amino acid homeostasis. Last, electrophysiological evidence in rats supports a hepatoportal amino acid sensing mechanism [90], but the functional consequences have not been identified.

Thus, dietary proteins and amino acids are sensed in the oral cavity and intestine, as well as by vagal and splanchnic afferents. These sensing modalities clearly modulate appetite and consummatory behaviours in response to changes in dietary protein intake. However, we still poorly understand how these sensing mechanisms might specifically contribute to organismal amino acid homeostasis

### 2.3. Brain sensing of systemic amino acid availability: fibroblast growth factor 21

Fibroblast growth factor 21 (FGF21) is a metabolic hormone initially identified as a fasting-induced signal that orchestrates adaptations to starvation [91]. It is primarily produced by the liver [92] but is also expressed in adipose tissue, skeletal muscle and the pancreas [93]. FGF21 serves as a key regulator of metabolism, influencing glucose uptake [94], insulin sensitivity, lipogenesis and brown fat thermogenesis [95]. FGF21 signals through a receptor complex composed of FGF receptors and the co-receptor β-klotho (KLB), which is present in peripheral tissues and certain brain regions, including the hypothalamus [96]. Many metabolic effects of FGF21 rely on brain signalling, which stimulates sympathetic output to fat and activates the stress axis [97]. Central FGF21 signalling has also been implicated in regulating sucrose intake, alcohol consumption, and dietary macronutrient preferences, and these effects are all supported by human genetic studies [98–102].

Research in rodents suggests that FGF21 acts as a signal of dietary protein restriction, enabling the brain to drive behavioural adaptations to amino acid deficiency. Plasma FGF21 levels significantly increase following dietary EAA deficiency or prolonged protein restriction in both humans and rodents. Under these conditions, FGF21 production is upregulated specifically in the liver following activation of the integrated stress response [103–105]. Pharmacological administration of FGF21 has been proposed to specifically enhance protein preference [106,107], though this remains debated with studies also showing an effect on alcohol or sucrose preference [102,108]. The effect of FGF21 on sweet preference has been proposed to depend on daily carbohydrate intake, a potential explanation for contradictory reports in the literature [107]. Mice lacking FGF21 or its brain receptor fail to show the typical responses to protein restriction, including hyperphagia, protein preference and motivation to work for protein [19,64,103,106]. Further studies point to glutamatergic neurons as mediators of the response to protein restriction, although the specific neuronal populations involved remain unclear [109]. This body of literature is convincing, but the following considerations are important to define the contexts in which FGF21 acts to signal dietary protein restriction.

Most of the mechanistic insights into the role of FGF21 in the response to protein restriction have been derived from studies using mice adapted to diets containing 5% or less dietary protein—essentially a model of protein malnutrition. Such diets rapidly disrupt growth, lead to a loss of lean mass, cause hypoalbuminemia and induce nutritional stress [110,111]. These observations suggest that at this level of protein restriction, organisms fail to adapt amino acid metabolism to compensate for the drastic reduction in dietary protein intake. Consistently, systemic amino acid homeostasis is impaired under these conditions [103]. This failure is perhaps not surprising considering that a 5% protein diet provides four times less protein than standard laboratory diets, requiring a fourfold increase in energy intake to offset dietary protein deficit through hyperphagia. For comparison, high-fat feeding only results in a 50% increase in daily energy intake [112], making this fourfold increase likely unachievable. Distinct signals, sensing mechanisms and adaptive metabolic responses might be engaged when animals successfully maintain systemic amino acid availability during milder protein restriction. Research models in which organisms successfully employ compensatory behavioural and metabolic strategies to address reduced dietary protein availability without compromising growth or lean mass might help identify these distinct signals and pathways to advance our mechanistic understanding of the homeostatic control of systemic EAA availability.

The potential role of FGF21 as a permissive signal facilitating central adaptations during protein deficiency should also be examined. FGF21 signalling integrates into a broader network of pathways regulating cellular metabolism, including amino acid sensing mechanisms such as mTORC1. This raises the possibility that deletion of FGF21 or its co-receptor KLB might constitutively maintain mTOR active, bypassing other mechanisms of cellular sensing of amino acids or related metabolites and masking their effects. Supporting this notion, FGF21 knockout (KO) mice exhibit metabolic features consistent with aberrant activation of anabolic pathways, such as increased lean mass [113,114], pancreatic islet hyperplasia [115] and hepatocellular carcinoma [116]. Furthermore, the absence of muscle FGF21 signalling has been shown to block the muscle atrophy response to protein restriction [114]. Interestingly, high-protein feeding leads to a marked reduction in hepatic and circulating FGF21 levels, further suggesting that FGF21 downregulation is part of the endocrine signature of the anabolic state [117]. Although the effects of neuronal Klb KO on intracellular metabolism in neurons remain poorly understood, this genetic manipulation might trigger similar cellular responses, potentially masking the effects of alternative sensing mechanisms. An experiment to clarify this would involve rescuing FGF21 signalling in FGF21 KO mice and assessing whether this intervention restores the adaptive responses to protein restriction. Pharmacological studies using FGF21 injections indicate that brain FGF21 signalling influences dietary protein-to-carbohydrate ratio without acutely affecting energy intake or expenditure, unlike what is observed during protein restriction [106,118,119]. In addition, hyperphagia is not reported in other models of protein deficiency with elevated FGF21 levels [120]. These findings leave unresolved whether brain FGF21 signalling directly and specifically promotes protein appetite or mediates the broader physiological adaptations to protein restriction.

Another important point is that our understanding of the early response to dietary protein restriction remains limited. In mice, FGF21 levels are significantly elevated after 4 days of consuming a 5% protein diet, and increased energy expenditure under these conditions also becomes evident after 4 days [103]. Alternative mechanisms may be at play during the acute phase of protein restriction, prior to the activation of FGF21. This raises the possibility that hepatic FGF21 activation might represent a secondary response to amino acid deficiency rather than a primary driver of downstream adaptations. Notably, primary hepatocytes do not upregulate FGF21 expression in response to amino acid starvation *in vitro* [121], highlighting the potential involvement of alternative regulatory mechanisms *in vivo*.

Last, FGF21 is induced under various nutritional conditions, including overfeeding and ketogenic diets [122], not consistently linked to increased protein preference. Interestingly, mice lacking UCP1 fail to exhibit both the hyperphagic and hypermetabolic responses to protein restriction. This suggests that UCP1-dependent thermogenesis and increased energy expenditure may represent primary responses to protein restriction, driving the associated hyperphagia [123]. At supra-pharmacological levels, the hypermetabolic response to exogenous FGF21 depends on total caloric intake, with FGF21 specifically increasing energy expenditure when mice are maintained on a higher energy diet [107]. However, in this study, elevated FGF21 failed to increase daily energy intake, regardless of the dietary caloric content. This leaves us short of a specific signal for systemic amino acid deficiency to mediate protein hunger and the associated metabolic adaptations under physiological conditions.

### 2.4. Brain sensing of systemic amino acid availability: direct brain amino acid sensing

Specialized nutrient-sensing neurons continuously monitor systemic nutrient availability and play an important role in maintaining nutrient homeostasis [124]. These neurons adjust their electrical activity in response to fluctuations in extracellular nutrient levels and activate downstream circuits to coordinate behavioural, autonomic and neuroendocrine feedback responses [124]. The role of brain nutrient sensing in the maintenance of systemic glucose homeostasis is well established (see [125] for a recent review). Similarly, brain amino acid sensing triggers feedback mechanisms, as first proposed by Panksepp and Booth, who observed that injecting a balanced amino acid solution into the hypothalamus suppresses food intake [126]. In this section, we review current insights into brain amino acid sensing mechanisms and their role in appetite regulation.

The brain is exposed to diet-induced changes in amino acid concentrations. This was first shown in rats, where marked increases in brain BCAAs and tyrosine concentrations were observed in the postprandial state, mirroring changes in circulating levels and amounts of ingested protein [127–130]. Microdialysis studies revealed region-specific changes [131–134] and consistent postprandial increases in extracellular levels of BCAAs in the lateral nucleus of the hypothalamus (LH), the paraventricular nucleus of the hypothalamus (PVH) and the medial preoptic area. Notably, brain amino acid levels return to baseline within 3 h after either feeding or intragastric infusion of amino acid mixture [131–134]. On the other hand, dietary protein restriction does not significantly alter brain BCAA and EAA levels despite the decrease of their circulating counterparts [135]. These observations support the existence of a buffering mechanism further stabilizing brain amino acid levels.

Systematic intracerebroventricular (ICV) injections of individual amino acids have demonstrated that only central administration of leucine consistently induces anorectic responses in rodents [136–138]. Targeted leucine injections into the mediobasal hypothalamus and the dorsovagal complex—two brain regions enriched in nutrient-sensing neurons—also suppress food intake. This is achieved through delayed meal initiation, acute reduction in meal size, and decreased meal frequency, leading to significant weight loss [138,139]. Thus, brain leucine sensing influences multiple components of feeding behaviour, suggesting the engagement of distinct appetite-regulating circuits.

These circuits do not include aversion pathways, as leucine sensing does not elicit conditioned taste avoidance [136,139]. Instead, leucine recruits pro-opiomelanocortin (POMC) neurons in the arcuate nucleus of the hypothalamus, as well as their projections to the PVH and the nucleus of the solitary tract (NTS), to reduce meal size [138]. A subset of NTS neurons also directly responds to local changes in leucine concentration [139]. These include NTS catecholaminergic neurons [139] and neurons co-expressing calcitonin receptor and prolactin-releasing peptide [140,141]. Using activity-dependent circuit mapping, we found that NTS leucine sensing inhibits hypothalamic AGRP neurons, and this ascending brainstem-to-hypothalamus circuit mediates leucine-induced changes in meal initiation and satiety [140,141]. Importantly, genetic silencing of this circuit blunts the anorectic effects of high-protein feeding [141], demonstrating its physiological relevance beyond the pharmacological paradigm that initially enabled its identification.

Mechanistically, leucine has been proposed to modulate electrical activity in leucine-sensing neurons in an mTOR-dependent manner [136,142,143]. However, leucine depolarizes POMC neurons in mouse hypothalamic brain sections [142], and this effect persists in brain sections from mice with inactive mTOR signalling [143], suggesting the recruitment of alternative mechanisms. Consistently, dissociated adult murine POMC neurons respond to leucine exposure within minutes in an mTOR-independent manner [144]. Using phosphoribosomal profiling of mediobasal hypothalamic neurons activated following local leucine injection, we identified the T-type voltage-gated calcium channel Cav3.1 as a marker of leucine-activated neurons [145]. Follow-up studies further revealed a direct role for Cav3.1 in neuronal leucine sensing. Cav3.1 is expressed in a neurochemically diverse population of hypothalamic neurons, including POMC neurons [145]. Leucine directly binds to the voltage-sensing transmembrane segment of Cav3.1, enhancing its sensitivity to voltage-dependent activation and leading to POMC neuron depolarization [145]. Pharmacological and genetic inhibition of Cav3.1 activity in hypothalamic POMC neurons blunts the anorectic response to central leucine administration. Importantly, genetic inhibition of Cav3.1 in POMC neurons also reduces the anorectic response to high-protein feeding, further supporting a physiological role for this sensing mechanism in dietary protein intake regulation [145].

The physiological relevance of brain leucine-sensing pathways and their control of feeding behaviour is likely restricted to the postprandial period, when leucine concentration in nutrient-sensing circuits is significantly elevated [142]. Consistently, several lines of evidence highlight important interactions with other meal-related signals. For example, NTS leucine sensing enhances the satiation response to CCK [146]. Additionally, the leucine sensor Cav3.1 physically interacts with and activates transient receptor potential channel 5 (TRPC5), thereby potentiating the electrical and anorectic responses to signals that depolarize POMC neurons via TRPCs, such as leptin, GLP-1 or serotonin [145,147]. These findings suggest that brain leucine sensing functions as an integrative mechanism that enhances the effects of various meal-related signals to regulate appetite.

Intriguingly, while the BCAA abundance has long been associated with satiety and energy repletion, a recent study demonstrated that mice fed on diets with high BCAA-to-non-BCAA ratio result in hyperphagia and increased weight gain [148]. The hyperphagic response to this amino acid imbalanced diet appears to be compensatory to reach the target intake of specific EAAs, akin to the hyperphagic feeding responses under a protein-restricted diet. The authors further identified that the blunted biosynthesis of a satiety-inducing neurotransmitter serotonin due to the reduced availability of its precursor tryptophan in the BCAA-enriched diet as an underlying mechanism [148]. Thus, this study highlights a context-dependent interaction between direct and indirect amino acid sensing of the brain to regulate energy homeostasis.

Little is known about the role of direct brain amino acid sensing in the regulation of feeding behaviour beyond canonical homeostatic circuits. An exception is the orexin/hypocretin neurons of the LH, which promote arousal, motivation and food intake and also respond to changes in amino acid concentrations (see [149] for a review). Interestingly, these neurons are specifically activated by non-essential amino acids (NEAAs) but not EAAs [150], leading to the suppression of feeding behaviour and the promotion of food-seeking [151]. The authors propose that this mechanism may have evolved to favour the ingestion of EAAs over NEAAs, ensuring an adequate intake of essential nutrients.

Strikingly, the available literature indicates that direct brain amino acid sensing does not contribute to hyperphagic response to low-protein feeding. To test the hypothesis that brain detection of hypoaminoacidemia might drive hyperphagia in protein-restricted rodents, Larger *et al.* used central infusion of amino acid mixtures in rats maintained on a low-protein diet [135]. This failed to alleviate the hyperphagic response to low-protein feeding, arguing against a role for brain amino acid sensing in this response. Of note, it appears that brain infusion of amino acid solution instead led to an apparent increase in food intake, especially during the early days of low-protein diet feeding, suggesting that the role of brain amino acid sensing in appetite control can be protein-state-dependent [135]. Also important to note, the constant brain infusion approach does not recapitulate the postprandial brain amino acid bioavailability profile and could lead to blunted sensitivity. The physiological role of brain amino acid sensing under protein restriction warrants further study.

In contrast, brain sensing of amino acid deficiency by the anterior piriform cortex (APC) plays a major role in the aversive responses to diets with an imbalanced composition of EAAs [66]. *Ex vivo* brain slice experiments revealed that APC glutamatergic neurons are directly activated by EAA depletion, causing tRNA uncharging and activation of the GCN2 (general control nonderepressible 2)–eIF2a signalling axis [152,153]. Subsequent studies showed that both whole body and CNS-specific GCN2-deficient mice fail to reject EAA-deficient diets, confirming the pivotal role of GCN2 in brain sensing of EAA deficiency [154,155]. However, these conclusions have been challenged by a recent study using global GCN2-deficient mice [156,157], although differences in experimental design might account for this discrepancy [158]. Together, these studies support a role for brain amino acid sensing in the avoidance of diets with imbalanced EAA composition.

Collectively, these studies indicate a role for direct brain amino acid sensing in the processing of meal-related increases in amino acid availability and the production of negative feedback controls of food ingestion. However, the converse is not true, with a lack of support for the recruitment of brain amino acid sensing circuits during hypoaminoacidemia. It is worth noting that in rodents, circulating concentrations of EAAs return to baseline 3 days after the introduction of a low-protein diet [135], and thus the brain is not exposed to changes in EAA availability under these conditions. Instead, amino acid metabolites might encode long-term changes in dietary amino acid availability. Several EAA-derived metabolites have been found to have potent effects on metabolism and appetite controls. For example, N-lactoyl phenylalanine (Lac-Phe), synthesized in intestinal epithelial cells, has been shown to mediate appetite suppression in both humans and mice through its central action [159,160]. Ketone body β-hydroxybutyrate, which modifies free amino acids via the α-amide group, suppresses food consumption via a central mechanism [161]. The relevance of these EAA derivatives in signalling protein status has yet to be established.

## 3. Brain amino acid sensing and the control of metabolic effectors for amino acid homeostasis

Adaptive changes in feeding behaviour in response to experimental conditions of low- or high-protein intake rarely enable the maintenance of dietary protein consumption at a fixed ‘target protein intake’ in rodents or humans. For example, a 50% reduction in dietary protein concentration leads to only a 13% increase in energy intake in healthy participants [57] and a 10% increase in mice [40,162]. Despite these adjustments, nitrogen balance is maintained [163], and post-absorptive levels of EAAs are rapidly normalized [135] under a variety of nutritional conditions associated with inadequate protein intake (table 1). This suggests that organisms can adapt to inadequate dietary protein intake and maintain the availability of circulating amino acids by modulating amino acid metabolism. In this section, we will briefly summarize the current understanding of the metabolic adaptations occurring in response to changes in dietary protein intake to maintain organismal amino acid homeostasis and explore the pathways through which the brain might modulate these responses.

### 3.1. Metabolic adaptations for organismal amino acid homeostasis

Splanchnic adaptations to changes in dietary protein intake play a significant role in buffering the impact of these changes on organismal amino acid availability. For instance, intestinal aminopeptidase activity shows a linear correlation with dietary protein intake [162,164]. Intestinal utilization of ingested amino acids is substantial and influenced by dietary protein quality, yet little is known about how this utilization adjusts in response to chronic variations in dietary protein concentration [165]. Similarly, hepatic amino acid metabolism undergoes significant adaptations, with the expression of several enzymes involved in amino acid oxidation positively correlating with dietary protein intake [20,164,166,167].

Skeletal muscle protein metabolism is also influenced by circulating amino acid levels, playing a key role in regulating postprandial BCAA availability and post-absorptive levels of gluconeogenic amino acids [168,169].

Last, brown adipose tissue has emerged as an important site for amino acid oxidation [28,170]. Increased amino acid availability has been suggested to activate adaptive thermogenesis in brown and beige adipocytes [171,172]. The contribution of this pathway to systemic amino acid homeostasis remains untested. The prediction would be that it might help buffer systemic amino acid levels for organismal amino acid homeostasis.

### 3.2. Brain amino acid sensing for the metabolic control of circulating amino acid availability

Evidence directly implicating brain amino acid sensing pathways in the control of peripheral amino acid metabolism is scarce. Hepatic gluconeogenesis is under sympathetic control [173] and is increased in response to hypothalamic leucine sensing or activation or hypothalamic leucine catabolism [174]. These interventions decrease hepatic glucose output, but the impact on circulating amino acid concentrations has not been reported. Hypothalamic leucine sensing and mTOR activity have also been implicated in the sympathetic control of brown fat thermogenesis [175]. Specifically, hypothalamic detection of increased leucine availability activates brown fat thermogenesis, while inhibition of hypothalamic mTORC1 with rapamycin produces the opposite effect. In conditions of increased circulating concentrations of amino acids, this feedback could promote BCAA disposal by local oxidation in the brown adipose tissue [28] and help restore systemic amino acid homeostasis. More work is needed to specifically test the role of brain amino acid sensing in the regulation of circulating amino acid metabolism.

In contrast, the role of brain insulin signalling in the regulation of circulating amino acid concentrations has been directly tested. Hypothalamic insulin injections dose-dependently increase hepatic BCAA catabolism [176] through the recruitment of vagal output [177], leading to reductions in circulating BCAA levels. Conversely, CNS-specific insulin signalling deficiency impairs systemic BCAA metabolism, leading to sustained elevation of circulating BCAA levels, supporting a critical role for the brain in organismal amino acid homeostasis [176].

The pancreatic hormone glucagon is also a major inducer of amino acid catabolism [178–180], important for the maintenance of systemic amino acid homeostasis [181–184]. The autonomic control of glucagon secretion in the context of the counter-regulatory response to hypoglycaemia is well established [185,186]. However, how brain amino acid sensing might recruit the parasympathetic nervous system to modulate glucagon secretion and promote its effects on amino acid clearance is unknown.

Neuroendocrine axes represent powerful mechanisms through which the brain influences peripheral physiology. Emerging evidence suggests that brain detection of amino acid deficits—via pathways such as mTOR and GCN2—regulates the output of the hypothalamic–pituitary–adrenal axis and the thyroid axis [187–189]. Glucocorticoids stimulate protein breakdown, releasing amino acids into circulation during stress or fasting. However, the relevance of these neuroendocrine pathways to the maintenance of amino acid homeostasis remains unclear. In addition, glucagon secretion is activated in response to arginine-vasopressin (AVP) release in the pituitary [190]. Of note, increased dietary protein intake increases water intake through an activation of vasopressin release [191,192]. Thus, the vasopressin axis responds to changes in peripheral amino acid availability and might contribute to the hyperglucagonemic response in this context.

In summary, brain amino acid-sensing pathways regulate neuroendocrine, sympathetic and parasympathetic effectors that control peripheral metabolism. These nutrient-sensing pathways in the brain can specifically influence peripheral amino acid utilization. However, further research is required to elucidate how these pathways are activated and coordinated to maintain organismal amino acid homeostasis.

## 4. Brain detection of amino acid deficit: a mechanism to prioritize physiological functions?

During prolonged reduction in dietary protein intake, growth and reproductive success are reduced or completely halted while nutritional resources are redirected to maintain vital physiological functions and for somatic cell maintenance [193–197]. The brain is recognized as a central regulator of the somatotropic and gonadotropic neuroendocrine axes, which provide feedback for the regulation of growth and reproduction. In this final section, we focus on the adaptations that occur under conditions of prolonged protein restriction. Rather than detailing the mechanisms behind these adaptations, we discuss the existing evidence that allows us to propose a role for brain sensing of amino acid deficit in these adaptations.

### 4.1. Brain amino acid sensing and the somatotropic axis

Growth hormone (GH) and insulin-like growth factor-1 (IGF-1) are pleiotropic hormones with crucial roles in growth, cell proliferation and differentiation, lifespan regulation and whole-body metabolism. GH is released from the pituitary gland and stimulates hepatic secretion of IGF-1 [198], mediating the peripheral effects of the somatotropic axis [199]. Regulation of the GH/IGF-1 axis by nutrient and energy availability is critical in the adaptation to starvation [200,201]. These effects include the regulation of peripheral amino acid fluxes, such as gluconeogenesis and muscle amino acid sparing [202]. In addition, GH treatment promotes protein appetite in rats, leading to greater protein accretion [203]. Thus, GH regulates behavioural and metabolic effectors of amino acid homeostasis.

Amino acids directly regulate GH release and IGF-1 concentrations [204–206], and this action is modulated in response to changes in dietary protein intake [207,208]. In rats maintained on a very low-protein diet (4%), a model of growth restriction, both spontaneous and GHRH-induced GH release are markedly reduced [209]. However, direct evidence linking brain amino acid sensing to this regulation is lacking. Hypothalamic GHRH neurons are known to be glucose-sensitive [210], but their ability to directly respond to fluctuations in amino acid levels remains unexplored.

In addition, GH release is regulated by a number of peripheral hormones including FGF21 [200], which mediates GH-induced growth inhibition under conditions of food restriction [211] and contributes to GH-induced reductions in lean mass [212]. After ICV administration of FGF21, reduced tibialis anterior muscle size is observed, which correlates with elevated plasma amino acid concentrations. This finding suggests that FGF21 may promote muscle wasting to maintain physiological amino acid levels during protein restriction [212].

### 4.2. Brain amino acid sensing and the reproductive axis

Hypothalamic gonadotropin-releasing hormone (GnRH) neurons regulate the hypothalamus–pituitary–gonadal (HPG) axis, which drives gamete formation and the release of sex hormones [213]. Kisspeptin (Kiss1) signalling is essential for the pulsatile release of GnRH, regulated by both stimulatory signals from neurokinin B and inhibitory signals from dynorphin A, both co-expressed with kisspeptin in KNDy neurons [213]. Emerging evidence highlights kisspeptin neurons as key integrators of interoceptive signals in regulating the HPG axis [214].

Clinical and rodent studies consistently demonstrate that dietary protein intake modulates puberty onset and reproductive function [193,196,215]. High-protein intake is generally associated with earlier puberty onset [196,216] and higher fertility potential in both males and females [217,218]. Conversely, protein restriction during periods of active growth and development delays sexual maturation and impairs reproductive function [219]. How protein status is integrated into the regulation of the HPG axis is unclear, but based on the effect of energy restriction on this neuroendocrine axis, we can speculate on the underpinning mechanisms.

GnRH neurons lack insulin and leptin receptors, suggesting that upstream neurons mediate the integration of metabolic and reproductive functions. In contrast, Kiss1 neurons express receptors for leptin [220], insulin [221] and IGF-1 [222] and interact directly with pro-opiomelanocortin (POMC) and AGRP neurons [223], which are key players in energy and amino acid sensing. Direct amino acid sensing within Kiss1 neurons may also regulate their output. Indeed, inhibition of mTOR signalling in Kiss1 neurons reduces Kiss1 expression, delays puberty onset and suppresses luteinizing hormone secretion [224]. In food-restricted female rats, central administration of leucine during puberty partially restored luteinizing hormone levels, doubling them compared to vehicle-treated food-restricted controls [224]. However, in ad libitum-fed prepubertal rats, leucine administration had no effect on luteinizing hormone levels, suggesting that further activation of mTOR does not influence puberty onset under normal nutritional conditions [224]. These findings highlight the critical role of BCAA signalling, particularly in undernutrition, in regulating puberty onset and reproductive function.

During protein restriction, increased FGF21 signalling likely contributes to the regulation of the HPG axis. Overexpression of FGF21 leads to defects in the HPG axis, while deletion of FGF21 receptor Klb in the forebrain restores ovulation and fertility in mice overexpressing FGF21 [225]. These findings suggest that elevated FGF21 contributes to the inhibition of the reproductive axis during protein restriction. Although Kiss1 neurons do not express Klb, FGF21 has been shown to influence Kiss1 expression, indicating an indirect mechanism through which FGF21 modulates reproductive function [221]. It is important to note, however, that the effect of FGF21 signalling on fertility seems to be specific for females. In further support of this observation, FGF21 selectively affects the population of AVPV kisspeptin neurons, which is absent in males [225].

Last, downregulation of GH release during protein restriction might contribute to the downregulation of the HPG axis in these conditions. The effect of GH signalling on the reproductive axis is highlighted by the consequences of the ablation of GH receptors in Kiss1 neurons or throughout the brain in pubertal mice, which significantly reduces Kiss1 and Gnrh1 expressions [199]. GH signalling also regulates sexual maturation and the ovulatory cycle [199]. Furthermore, deletion of IGF-1 receptors specifically in Kiss1 neurons results in reduced circulating testosterone levels [222]. These findings collectively suggest an additional mechanism through which changes in neuroendocrine responses during protein restriction might contribute to the downregulation of the reproductive axis.

## 5. Conclusion

In this review, we have highlighted experimental findings supporting the notion that circulating concentrations of EAAs are homeostatically regulated and examined the role of brain amino acid sensing in controlling behavioural and metabolic effectors that may be recruited to maintain this homeostatic regulation. The role of brain amino acid sensing in appetite responses is well established, but further research is needed to investigate its role in driving metabolic responses that help maintain circulating amino acid levels. Furthermore, while it is clear that systemic EAA availability is not maintained in severe models of protein restriction in rodents, moderate restriction paradigms associated with successful maintenance of EAA availability without compromise on growth and lean mass should be further investigated.

## Data Availability

This article has no additional data.
